# Supplementary material for: Potential Benefits of Selenium Supplementation in Reducing Insulin Resistance in Patients with Cardiometabolic Diseases: A Systematic Review and Meta-Analysis
Source: Nutrients. 2022 Nov 21;14(22):4933. doi: 10.3390/nu14224933 (PMC9693215; doi:10.3390/nu14224933)
Supplement: Supplementary file 1 [file nutrients-14-04933-s001.zip › nutrients-2001701-supplementary.pdf]

Supplementary Materials

Table S1. Search strategy for each electronic database.

Database	Strategy
**PubMed** (title/abstract)	#1 (selenium) OR (selenite) OR (selenate) OR (Selenium supplementation) OR (trace element)<br>#2 (cardiometabolic diseases) OR (diabetes mellitus) OR (type 2 diabetes mellitus) OR (T2DM) OR (coronary heart disease) OR (ischemic heart disease) OR (heart failure) OR (Cardiac Failure) OR (hypertension) OR (hyperlipidemia) OR (metabolic syndrome) OR (nonalcoholic fatty liver disease) OR (NAFLD) OR (stroke) OR (obesity) OR (obese)<br>#3 (Randomized Controlled Trial) OR (RCT) OR (random*)<br>#4 #1 AND #2 AND #3
**Cochrane Library** (title/abstract/keyword)	#1 (selenium) OR (selenite) OR (selenate) OR (Selenium supplementation) OR (trace element)<br>#2 (cardiometabolic diseases) OR (diabetes mellitus) OR (type 2 diabetes mellitus) OR (T2DM) OR (coronary heart disease) OR (ischemic heart disease) OR (heart failure) OR (Cardiac Failure) OR (hypertension) OR (hyperlipidemia) OR (metabolic syndrome) OR (nonalcoholic fatty liver disease) OR (NAFLD) OR (stroke) OR (obesity) OR (obese)<br>#3 (Randomized Controlled Trial) OR (RCT) OR (random*)<br>#4 #1 AND #2 AND #3
**Embase** (title/abstract/keyword)	#1 "selenium" OR "selenite" OR "selenate" OR "Selenium supplementation" OR "trace element"<br>#2 "cardiometabolic diseases" OR "diabetes mellitus" OR "type 2 diabetes mellitus" OR "T2DM" OR "coronary heart disease" OR "ischemic heart disease" OR "heart failure" OR "Cardiac Failure" OR "hypertension" OR "hyperlipidemia" OR "metabolic syndrome" OR "nonalcoholic fatty liver disease" OR "NAFLD" OR "stroke" OR "obesity" OR "obese"<br>#3 "Randomized Controlled Trial" OR "RCT" OR "random*"<br>#4 #1 AND #2 AND #3
**Scopus** (title/abstract/keyword)	#1 "selenium" OR "selenite" OR "selenate" OR "selenium supplementation" OR "trace element"<br>#2 "cardiometabolic diseases" OR "diabetes mellitus" OR "type 2 diabetes mellitus" OR "t2dm" OR "coronary heart disease" OR "ischemic heart disease" OR "heart failure" OR "cardiac failure" OR "hypertension" OR "hyperlipidemia" OR "metabolic syndrome" OR "nonalcoholic fatty liver disease" OR "NAFLD" OR "stroke" OR "obesity" OR "obese"<br>#3 "randomized controlled trial" OR "RCT" OR "random*"

	#4 #1 AND #2 AND #3
**Web of Science**<br>(title/abstract/keyword)	#1 selenium OR selenite OR selenate OR Selenium supplementation OR trace element<br>#2 cardiometabolic diseases OR diabetes mellitus OR type 2 diabetes mellitus OR T2DM OR coronary heart disease OR ischemic heart disease OR heart failure OR Cardiac Failure OR hypertension OR hyperlipidemia OR metabolic syndrome OR nonalcoholic fatty liver disease OR NAFLD OR stroke OR obesity OR obese<br>#3 Randomized Controlled Trial OR RCT OR random*<br>#4 #1 AND #2 AND #3

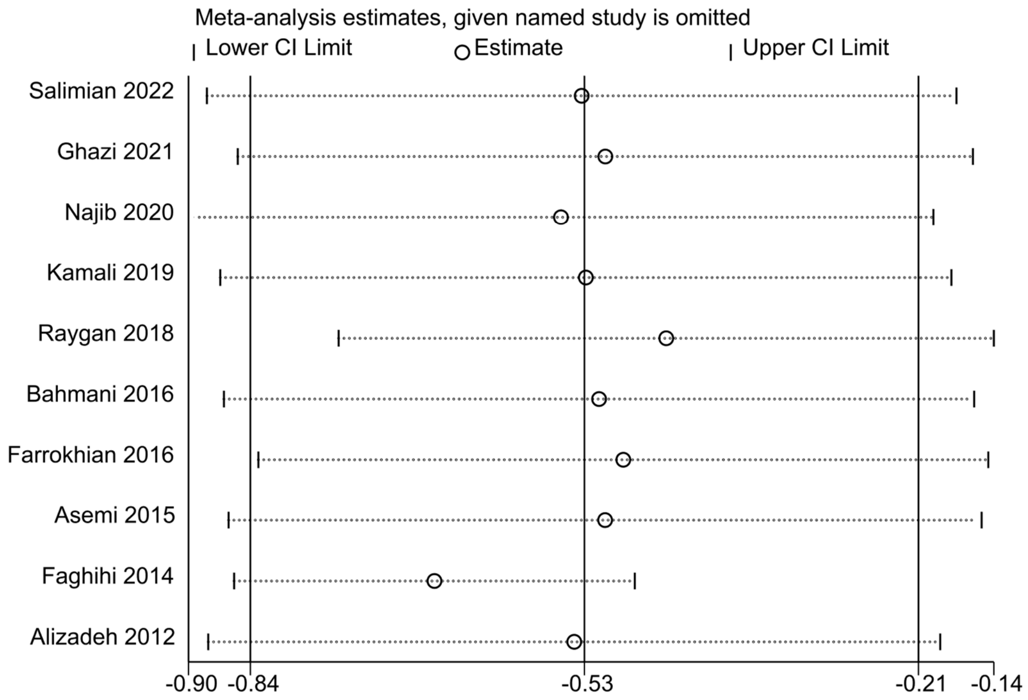

Figure S1. Sensitivity analysis of insulin levels.

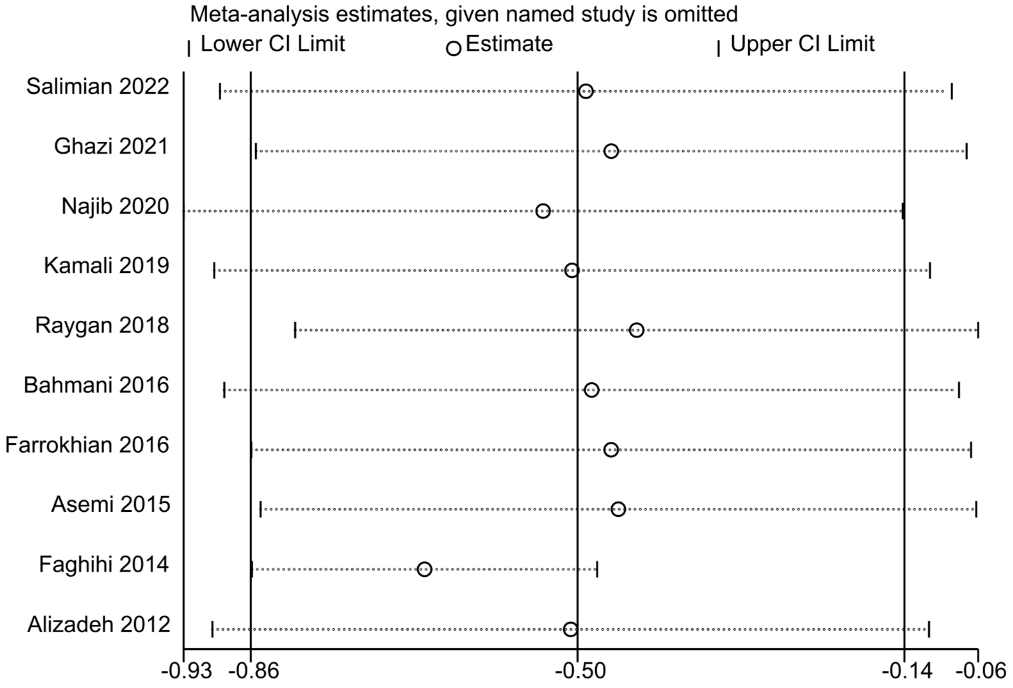

Figure S2. Sensitivity analysis of HOMA-IR.

	Selenium			Control				Std. Mean Difference
Study or Subgroup	Mean	SD	Total	Mean	SD	Total	Weight	IV, Random, 95% CI
Salimian 2022	-4.4	14.9	26	0.4	8	27	11.2%	-0.40 [-0.94, 0.15]
Ghazi 2021	-3.29	7.09	32	-0.11	8.7	17	11.0%	-0.41 [-1.00, 0.19]
Najib 2020	3.51	1.22	26	-3.52	3.53	28	10.5%	2.58 [1.85, 3.32]
Kamali 2019	-4.2	20.22	17	2.4	13.29	16	10.7%	-0.37 [-1.06, 0.32]
Raygan 2018	-0.42	1.01	26	0.07	1.07	27	11.2%	-0.46 [-1.01, 0.08]
Bahmani 2016	0.7	40.7	30	4.7	45.1	30	11.4%	-0.09 [-0.60, 0.41]
Farrokhian 2016	-2.2	58.5	30	-7.3	35.3	30	11.4%	0.10 [-0.40, 0.61]
Asemi 2015	-10.55	11.94	35	4.51	12.9	35	11.3%	-1.20 [-1.71, -0.69]
Faghihi 2014	16.34	35.45	33	-20.04	43.4	27	11.3%	0.92 [0.38, 1.45]
**Total (95% CI)**			**255**			**237**	**100.0%**	**0.06 [-0.56, 0.68]**

Heterogeneity: $Tau^2 = 0.82$; $Chi^2 = 87.51$, df = 8 ($P < 0.00001$); $I^2 = 91\%$
Test for overall effect: Z = 0.18 (P = 0.86)

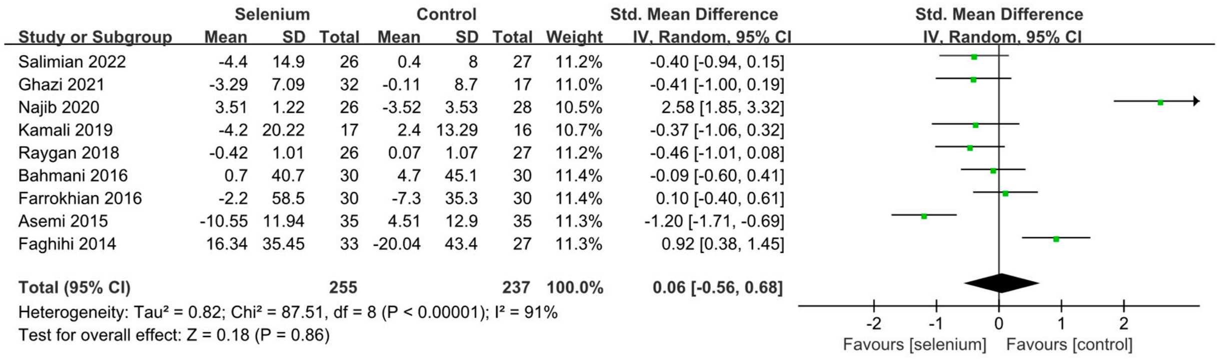

Figure S3. Forest plot of FPG.

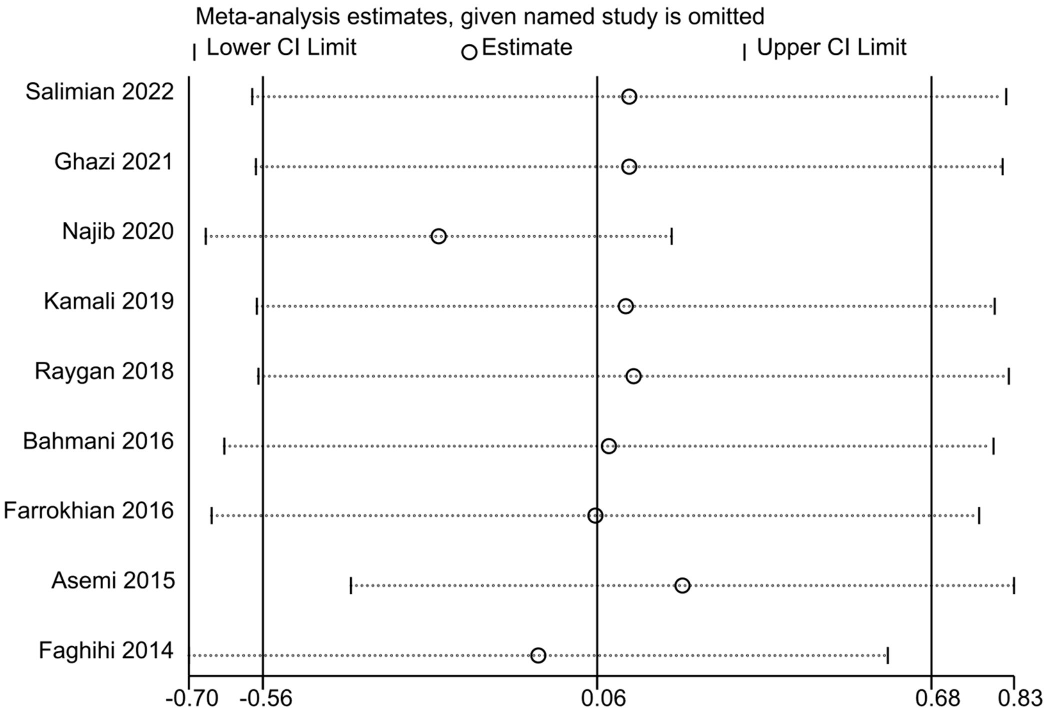

Figure S4. Sensitivity analysis of FPG.

Study or Subgroup	Selenium Mean	Selenium SD	Selenium Total	Control Mean	Control SD	Control Total	Weight	Std. Mean Difference IV, Random, 95% CI
Salimian 2022	2.5	2.7	26	0.2	1.2	27	11.9%	1.09 [0.51, 1.67]
Ghazi 2021	-0.22	0.17	32	-2.05	0.02	17	4.4%	13.00 [10.25, 15.74]
Kamali 2019	2.1	5.86	17	-0.7	5.72	16	11.5%	0.47 [-0.22, 1.16]
Raygan 2018	0.18	0.19	26	0.02	0.13	27	11.9%	0.97 [0.40, 1.54]
Bahmani 2016	0.4	4.1	30	-1.8	5.5	30	12.1%	0.45 [-0.07, 0.96]
Farrokhian 2016	1.3	7.7	30	1.2	6	30	12.1%	0.01 [-0.49, 0.52]
Asemi 2015	0.79	5.53	35	0.33	8.51	35	12.3%	0.06 [-0.41, 0.53]
Faghihi 2014	3.61	11.91	33	-1.84	9.47	27	12.1%	0.49 [-0.02, 1.01]
Alizadeh 2012	-0.5	6.23	17	0.7	5.41	17	11.6%	-0.20 [-0.87, 0.47]
**Total (95% CI)**			**246**			**226**	**100.0%**	**0.97 [0.26, 1.68]**

Heterogeneity: Tau² = 1.01; Chi² = 97.07, df = 8 (P < 0.00001); I² = 92%
Test for overall effect: Z = 2.69 (P = 0.007)

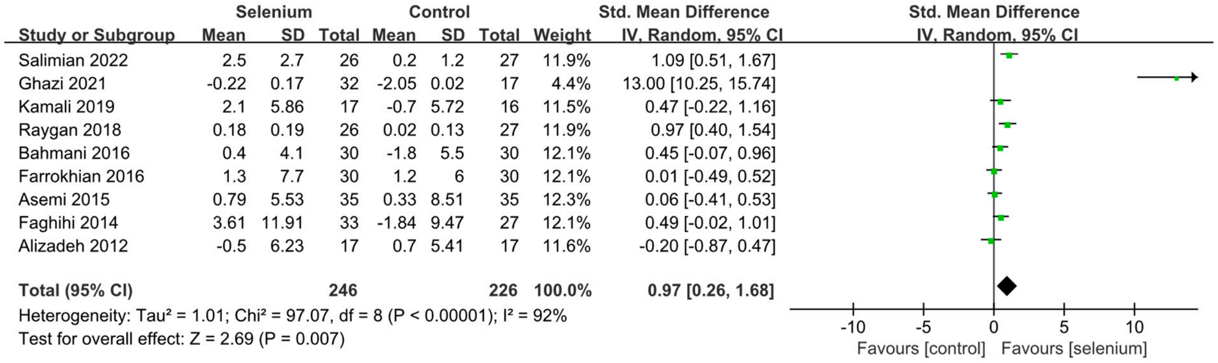

Figure S5. Forest plot of HDL-C.

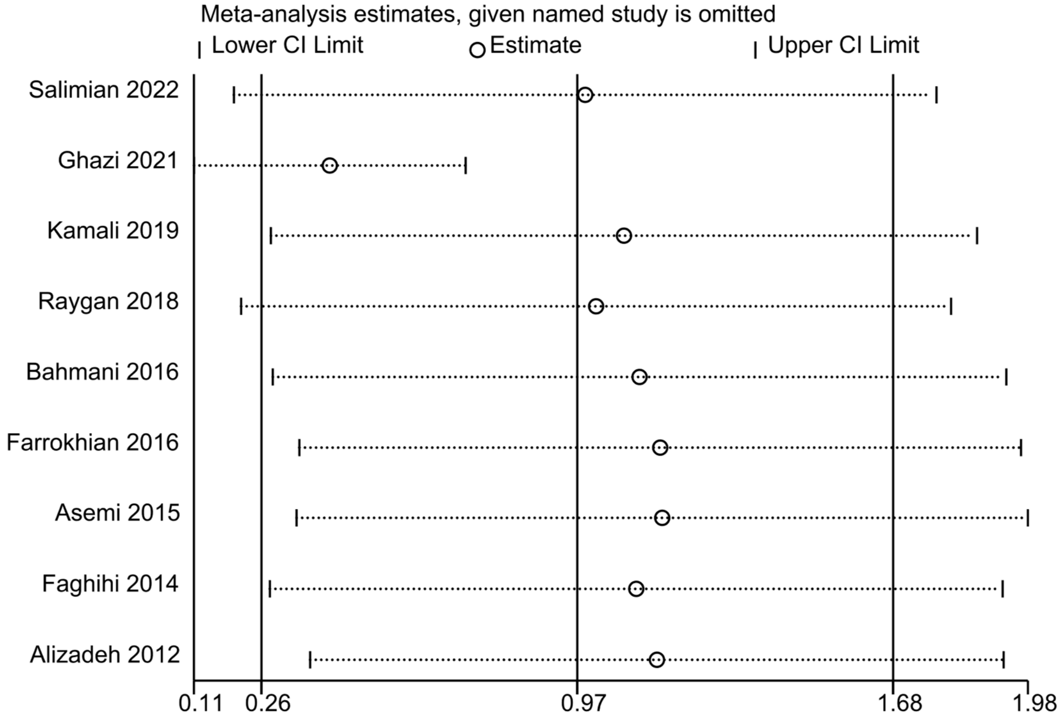

Figure S6. Sensitivity analysis of HDL-C.

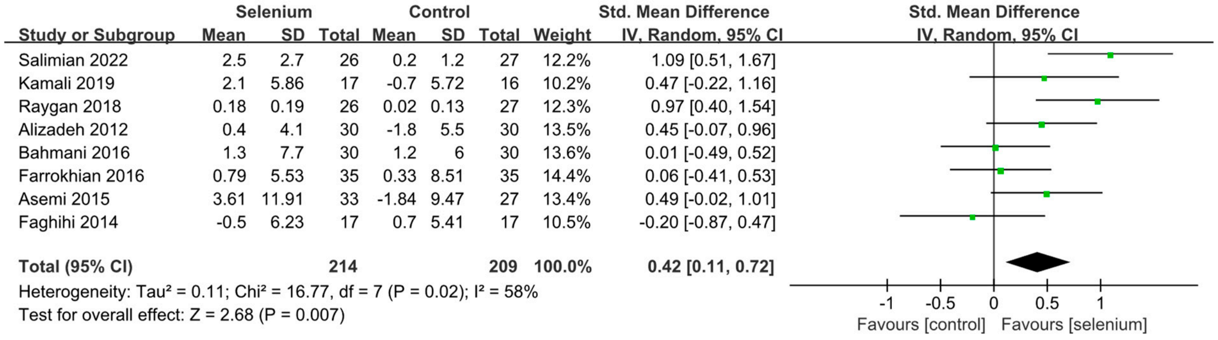

Study or Subgroup	Selenium Mean	Selenium SD	Selenium Total	Control Mean	Control SD	Control Total	Weight	Std. Mean Difference IV, Random, 95% CI
Salimian 2022	2.5	2.7	26	0.2	1.2	27	12.2%	1.09 [0.51, 1.67]
Kamali 2019	2.1	5.86	17	-0.7	5.72	16	10.2%	0.47 [-0.22, 1.16]
Raygan 2018	0.18	0.19	26	0.02	0.13	27	12.3%	0.97 [0.40, 1.54]
Alizadeh 2012	0.4	4.1	30	-1.8	5.5	30	13.5%	0.45 [-0.07, 0.96]
Bahmani 2016	1.3	7.7	30	1.2	6	30	13.6%	0.01 [-0.49, 0.52]
Farrokhian 2016	0.79	5.53	35	0.33	8.51	35	14.4%	0.06 [-0.41, 0.53]
Asemi 2015	3.61	11.91	33	-1.84	9.47	27	13.4%	0.49 [-0.02, 1.01]
Faghihi 2014	-0.5	6.23	17	0.7	5.41	17	10.5%	-0.20 [-0.87, 0.47]
**Total (95% CI)**			**214**			**209**	**100.0%**	**0.42 [0.11, 0.72]**

Heterogeneity: Tau² = 0.11; Chi² = 16.77, df = 7 (P = 0.02); I² = 58%
Test for overall effect: Z = 2.68 (P = 0.007)

Figure S7. Forest plot of HDL-C after excluding Ghazi 2021.
